# GlycoBioinformatics

**DOI:** 10.3762/bjoc.17.184

**Published:** 2021-11-09

**Authors:** Kiyoko F Aoki-Kinoshita, Frédérique Lisacek, Niclas Karlsson, Daniel Kolarich, Nicolle H Packer

**Affiliations:** 1 Faculty of Science and Engineering, Soka University, 1-236 Tangi-machi, Hachioji-shi, Tokyo, Japan; 2 University of Geneva and Swiss Institute of Bioinformatics, CUI - 7, route de Drize, 1211 Geneva, Switzerland; 3 Department of Medical Biochemistry and Cell Biology, University of Gothenburg, Box 440, 40530 Gothenburg, Sweden; 4 Faculty of Health Sciences, Department of Life Sciences and Health, Pharmacy, Oslo Metropolitan University, 0167 Oslo, Norway; 5 Griffith University, Gold Coast Campus, Southport, Queensland 4222, Australia; 6 Department of Molecular Sciences, Macquarie University, Sydney, New South Wales, Australia

**Keywords:** bioinformatics, glycobioinformatics, glycoinformatics

In order to introduce this thematic issue “GlycoBioinformatics” [1] in the *Beilstein Journal of Organic Chemistry*, it would be appropriate to define what we actually mean by this term. This is important not only for newcomers to the field but also in order for researchers that have used or developed “glycobioinformatics” to place their work into a wider context of this diverse field. The term “bioinformatics” is described by the National Human Genome Research Institute as “a subdiscipline of biology and computer science concerned with the acquisition, storage, analysis, and dissemination of biological data, most often DNA and amino acid sequences”. Adding the prefix “glyco-“ is about placing genomic and proteomic data into a glycomic context by harvesting information about glyco-related genes and proteins. Glycobioinformatics requires additional information about the expressed glycan, including but not limited to monosaccharide composition, full or partial sequence including linkage and branching structure, type and linkage of glycoconjugate (e.g., N-linked, O-linked glycoprotein, glycolipid, proteoglycan), association with, and regulation of, expression in particular tissues or cell types, and interaction with biological surroundings. With this definition, it is obvious that glycobioinformatics is tightly connected to mainstream bioinformatics. For example, databases and tools from genomics can be used for gaining information about genes encoding for glycosyltransferases, glycosidases, and glycan-binding proteins (lectins), and search engines initially designed for the detection of posttranslational modifications of peptides in proteomics can be adapted to specifically identify glycopeptides. What is also obvious for glycobioinformatics is that it needs an own language that is understood by both computers and researchers to facilitate the exchange of glyco-specific information as well as the development and evolution of dedicated databases that store glyco-related quality information. With glycobioinformatics still being in its infancy, these requirements are continuing to evolve.

The editors of this thematic issue represent both bioinformatics developers as well as users who have the conviction that important life science questions more often than not include an element of “glyco”. For this thematic issue, we have assembled publications from world-renowned glycoscience researchers who are involved in the current state-of-the-art glycobioinformatics approaches that are needed to find solutions for current global health challenges and to understand just about every biological process.

Molecular dynamic modeling to understand how glycans interact with biomolecules visualizes and allows the development of hypotheses regarding the function of glycans to be tested at a molecular level. The article by Barnett et al. [2] uses molecular dynamics to show that O-linked glycosylation alters peptide conformation, which influences the binding of the peptides to antibodies, despite the fact that glycans are not directly involved in the binding. Another molecular modeling article by Fogarty et al. [3] suggests a new concept of glycoblocks, which are subunits of 3D glycan structures. This concept may become useful in describing specific epitopes and functional units of glycans. With the recent pandemic experience, the need for glycobioinformatics for global health was highlighted, where the laboratory of one of the authors of this article, Fadda, used glyco-adapted molecular dynamics to explain in a separate publication [4] how the COVID-19 spike protein recognition element requires N-linked glycosylation to be exposed. Another approach to understanding glyco-interactions is described in a review paper by Mehta et al. [5], who summarize recent developments and available online resources for glycan array data, a very powerful technique for understanding the structural element(s) of glycans required for different lectin binding. This further emphasizes the role of glycans as mediators of cellular communication.

For newcomers and experienced glycoscience researchers, the review by Lal et al. [6] is a helpful guide to resources currently available for displaying glycan structures in 2D and 3D for scientific publications and presentations. The evolution of the “glyco” language is illustrated by Kellman et al. [7], wherein glycan substrate specificities and glycoenzyme reaction rules are described using an improved linear code that is standardized for use in analytical computational tools. This links with McDonald and Davey’s paper [8], which expands on their previously described theoretically derived protein O-linked glycome based on the specificity of mammalian glycoenzymes, in order to generate a theoretical glycolipid glycome.

One of the main tasks of glycobioinformatics is to convert analytical data obtained from biological samples (cell lysates, tissues, isolated proteins) into glycoscience knowledge. Most structural data at this stage is generated by analytical approaches, such as mass spectrometry (MS), high-pressure liquid chromatography (HPLC), and capillary electrophoresis (CE). The articles by Phung et al. [9] and by Lippold et al. [10] suggest ways of combining and customising available MS data analysis tools for glycoproteomic characterization and quantification. The article by Walsh et al. [11], on the other hand, addresses the problems of an irreproducible retention time and peak integration in antibody glycomic analysis using CE, thus allowing small quantitative differences to be detected when comparing similar glycomes by this method.

The articles by Groth et al. [12] and by Bagdonas et al. [13] illustrate how glycoinformation can be harvested and integrated from available -omics databases, with the former paper identifying putative cell signaling molecules and transcription factors using next-generation sequencing expression data of glycoenzymes in cancer cell lines. The latter paper uses knowledge from current open access glycomic databases to curate and validate glycan structures reported on proteins in the Protein Data Bank (PDB) database.

Overall, the wide breadth of glycobioinformatics articles that comprises this special issue only captures a snapshot of the impact that glycosciences and glycobioinformatics is now having across diverse scientific fields. These exciting results indicate the great progress that has been made and illustrates the huge potential for novel developments being made in this rather newly recognized field of life sciences.

Kiyoko F. Aoki-Kinoshita, Frédérique Lisacek, Niclas Karlsson, Daniel Kolarich and Nicolle H. Packer

Tokyo, Geneva, Gothenburg, Southport, Sydney, October 2021
